# Prescriptions and Formulæ

**Published:** 1877-04-20

**Authors:** 


### PRESCRIPTIONS AND FORMULÆ

VOMITING IN PREGNANCY.

For this troublesome affection a writer in Medical and Surgical Reporter states that he used the following remedy successfully for five years:

R—Pot. bromide...............dr. iij;

Tinc. columbo..............dr. ij;

Aquae.......................oz. ij. M.

S. Give a teaspoonful every three hours.

Dr. Chandler, in Louisville Medical News, submits the following:

CHILL TONIC.

R—Chinoidine.................oz. ss;
Subcarb iron. .............dr. ij;
Aloes.........................dr. ij;
Capsicum....................dr. j;
Fowler's solution.......fl. oz. j;
Whisky........................oz. vij. M.

S. Take a tablespoonful three times a day of the mixture, and continue for a fortnight.

ATONIC DYSPEPSIA AND CARDIALGIA.

R—Yellow-root...............oz. ij;
Nitrate of potash.........oz. j;
Willow charcoal...........oz. ss.

M. Take a teaspoonful of the powder in half a glass of water before meals; also

R—Tinct. iron muriate.........oz. j;
Tinct. nux vomica.........oz. j.

M. Ten to fifteen drops after meals. Regulate the quantity and quality of diet; prescribe hours of recreation, cheerful company at meals, and attempt to divert the mind of patient as much as possible from his malady during meals and the times of digestion.

AMENORRHEA IN ANÆMIC SUBJECTS.

R—Sulph. iron................dr. jss;
Guaiacum.................dr. ij;
Myrrh.......................dr. j;
Gentian.....................dr. ij;
Aloes.......................dr. ij;
Blood-root................dr. ss;
Whisky.....................pt. ij.

M. Dessertspoonful three times a day; also apiol drops v every three or four hours during the expected menstrual period and for some few days previous. This, with warm coxæluvia morning and night to invite the menstrual molimen, will seldom disappoint the practitioner in its results.

HOOPING COUGH.

R—Croton chloral hydrate...gr. xl;
Tinct. belladonnæ........fl. oz. ss;
Syrup pruni virg .......fl. oz. vss.

M. Teaspoonful three times a day for a child five years of age; to be increased or diminished with the exigencies of the case, the persistency of the attack, the age of the patient, etc.

COMMON COUGH MIXTURE.

R—Morphiæ sulphatis............gr. iij;
Acid hydrocyanici dil...fl. dr. ij;
Syr. pruni, virg...........fl. oz. vj.

M. S. Teaspoonful every four to six hours.

FOR SCROFULA.

R—Indian hemp.................oz. j;
Sarsaparilla...................oz. j;
Burdock......................oz. j;
Poke-root......................dr. j.

Add half a gallon of water and reduce by boiling to about a pint; strain and add one pound of sugar; reduce to one pint and add

R—Iod. potass..................oz. ss;

Citrate iron................oz, ss.

Of this mixture give a teaspoonful three times a day to a child ten or fifteen years of age.

CATARRH OF THE BLADDER.

R—Acetate potash.............dr. ijss;
Bromide potassium........dr. ijss;
Tinct. hyoscyamus........fl. oz. j;
Infus. buchu....................pt. j.

M. Dose, two ounces every three to six hours.

CHRONIC RHEUMATISM.

R—Guaiacum ...................oz. j;
Black cohosh................dr. ij;
Blue cohosh.................dr. ij;
Rhubarb.......................dr. j;
Wine colchicum........fl. oz. ij;
Iodi. potassium........... oz. ss;
Whisky......................pt. ij.

M. Dessertspoonful three times a day.

R—Camphor.....................oz. j;
Chloral hydrate...........oz. ss;
Chloroform.................oz. jss;
Soap liniment............oz. ijss.

M. Make a liniment. S. Use locally for the relief of pain.

